# Burden of Serious Bacterial Infections and Multidrug-Resistant Organisms in an Adult Population of Nepal: A Comparative Analysis of Minimally Invasive Tissue Sampling Informed Mortality Surveillance of Community and Hospital Deaths

**DOI:** 10.1093/cid/ciab773

**Published:** 2021-12-15

**Authors:** Suraj Bhattarai, Binita Koirala Sharma, Nuwadatta Subedi, Sunita Ranabhat, Madan Prasad Baral

**Affiliations:** 1 DECODE-MAUN Research Project, GMCTHRC, Pokhara, Nepal; 2 Department of Global Health, Global Institute for Interdisciplinary Studies, Kathmandu, Nepal; 3 Department of Microbiology, GMCTHRC, Pokhara, Nepal; 4 Department of Microbiology, Tribhuvan University Prithvi Narayan Campus, Pokhara, Nepal; 5 Department of Forensic Medicine, GMCTHRC, Pokhara, Nepal; 6 Department of Pathology, GMCTHRC, Pokhara, Nepal; 7 Department of Forensic Medicine, Pokhara Academy of Health Sciences, Western Regional Hospital, Pokhara, Nepal

**Keywords:** antimicrobial resistance, bacterial infections, cause of death, invasive bacterial disease, multidrug resistance

## Abstract

**Background:**

Bacterial diseases are the leading cause of mortality globally, and due to haphazard use of antibiotics, antimicrobial resistance has become an emerging threat.

**Methods:**

This cross-sectional observational study utilized a minimally invasive tissue sampling procedure to determine the cause of death among an adult population. Bacterial cultures (blood, cerebrospinal fluid, lung tissue) and antibiotic susceptibility were evaluated, and the results were compared between community and hospital deaths.

**Results:**

Of 100 deceased persons studied, 76 (76%) deaths occurred in the community and 24 (24%) in the hospital. At least 1 bacterial agent was cultured from 86 (86%) cases; of these, 74 (86%) had a bacterial disease attributed as the primary cause of death, with pneumonia (35, 47.3%), sepsis (33, 44.6%), and meningitis (3, 4.1%) most common. Of 154 bacterial isolates (76.6% from the community and 23.4% from the hospital) detected from 86 culture-positive cases, 26 (16.8%) were multidrug-resistant (MDR). *Klebsiella* species were the most common (13 of 26) MDR organisms. The odds of getting an MDR *Klebsiella* infection was 6-fold higher among hospital deaths compared with community deaths (95% confidence interval [CI], 1.37–26.40; *P* = .017) and almost 23-fold higher (CI, 2.45–213.54; *P* = .006) among cases with prior antibiotic use compared to those without.

**Conclusions:**

High incidence of serious bacterial infections causing death of adults in the community, with most MDR organisms isolated from hospitalized cases, calls for robust surveillance mechanisms and infection prevention activities at the community level and evidence-driven antibiotic stewardship in healthcare settings.

The burden of infectious diseases is high globally, more so in low- and lower-middle income countries (LMICs) compared with upper-middle and high-income countries where noncommunicable diseases are responsible for the majority of deaths [1, 2]. Approximately 7 million people died of infectious diseases in 2019, accounting for nearly 12% of all deaths globally [3]. Furthermore, it has been estimated that 700 000 deaths occur each year globally due to drug-resistant organisms, and the number may rise to 10 million with economic loss of $100 trillion by 2050, with higher impact in LMICs, if preventive and control measures are not taken [4]. The global landscape of disease burden, as well as ranking of infectious disease, may change due to the ongoing coronavirus disease 2019 (COVID-19) pandemic.

Although *Streptococcus pneumoniae*, *Haemophilus influenzae* type b, and *Neisseria meningitidis* (meningococcus) are the leading causes of invasive bacterial diseases in the pediatric population, the list of bacterial pathogens may differ in adults [4-6]. *Klebsiella pneumoniae*, *Acinetobacter* species, *Pseudomonas aeruginosa*, *Enterobacter* species, *Enterococcus faecium*, and *Staphylococcus aureus* are the major causes of morbidity and mortality in adults [7]. Moreover, drug-resistant organisms, particularly methicillin-resistant *S. aureus*, extended-spectrum β-lactamase (ESBL)–producing or carbapenemase-producing *K. pneumoniae*, and carbapenem-resistant or polymyxin-resistant *P. aeruginosa* and *Acinetobacter* species, have become a threat to the survival of infected adults in both community and healthcare settings [7-9].

Traditionally, the burden of infectious disease has been determined by 2 measures: incidence and mortality rates. However, there may be other short-term and long-term consequences of infectious disease at both individual and community levels that need to be explored, such as the alarming rise in antimicrobial resistance (AMR) and excessive burden to already fragile health systems, compromising quality of care [10]. Among these consequences, AMR is an emerging but the most threatening public health problem globally, considering a slow pace of new drug development [7, 11]. There is a great challenge to expand appropriate antimicrobial access along with restriction of incorrect usage in global health, especially to more expensive and newer-generation antimicrobials. This necessitates new approaches to financing and providing healthcare, as well as the One Health concept on transmission of pathogens in animals and humans [9].

Despite the high burden of bacterial diseases in Nepal, there is a paucity of quality data and evidence that can guide optimal clinical care and improve health outcomes of the patients. The country lacks robust surveillance mechanisms and longitudinal clinical research, which are needed to understand the real picture of bacterial infections and the impact of multidrug-resistant organisms (MDROs). Such evidence, if generated, would not only inform infection prevention policies but also help stakeholders design and estimate the cost of targeted interventions such as hygiene, sanitation, vaccination, and antibiotic stewardship [12, 13]. In Nepal, the burden of serious bacterial infections among children has been estimated, but not so in the adult population [14, 15].

Determining Efficiently the Cause of Death among Adults and Generating Mortality Evidence at MITS Alliance Unit Nepal is the first-ever mortality surveillance research in Nepal that collected clinical specimens through minimally invasive tissue sampling (MITS) and performed histopathological and microbiological tests to determine the cause of death [16]. In the current substudy, we aimed to estimate the burden of bacterial infections in the adult population, measured in terms of mortality attributable to a particular infection. We also aimed to know the profile of bacterial isolates causing infections and risk factors associated with multidrug resistance, compared between community and hospital deaths.

## METHODS

### Study Sites, Study Design, and Population

A quantitative, cross-sectional, observational study was conducted in parallel at 3 sites: Gandaki Medical College Teaching Hospital, Pokhara Academy of Health Sciences, and Damauli Hospital, all located in Gandaki Province of Nepal. These centers provide health services to around 10 million people in the province. A convenience sampling technique was used to enroll cases at the study sites during the study period (October 2019 to February 2021). The closest relatives of the deceased available at the site were approached for the consent process. The adult population (aged ≥18 years) with natural deaths (attributed to diseases and conditions) were included, whereas unnatural deaths (homicide, suicide, trauma, known case of poisoning) and perinatal deaths were excluded. Community death was defined as death that occurred at home or when a deceased individual was brought to the hospital; hospital death was defined as death that occurred within the hospital premises (emergency, in-patient, or critical care units) while the patient was undergoing treatment. Hospital deaths and the patients who died on the way to the hospital, but without having indication for forensic autopsy, were considered clinical cases, whereas those deaths that required mandatory forensic autopsy per the prevailing laws of the country were considered forensic cases.

### Clinical Data Collection

A standard questionnaire was used to collect clinical data using 2 approaches: verbal autopsy (interview in a structured proforma) with the family members and relatives of the study case and reviewing prior clinical records (if available) for deaths that were listed in the community and hospital records in case of hospital death [17]. The preexisting medical conditions of the study cases were also recorded in the case report form per the list of Charlson comorbidities, which include 17 diseases with different weights, ranging from myocardial infarction, peptic ulcer disease, and chronic obstructive pulmonary disease to moderate/severe liver disease and metastatic solid tumor [18].

### Specimen Collection

The MITS procedure was performed on the body of study cases at the morgue of study sites. For the clinical cases, the interval of time of death to MITS sampling was 2 hours on average. For the cases that had to undergo legal formalities for forensic autopsy, the bodies were kept in the cooling chamber at 4°C in the hospital morgue, and MITS was done within 72 hours of death. Twenty milliliters of blood was collected by supraclavicular approach or from the heart, 10 mL of cerebrospinal fluid (CSF) was collected from occipital puncture, and tissue specimens were biopsied from key organs using sterile Bard Monopty needles. A portion of lung and other tissue samples were sent for histopathological examination (pathology findings of clinical specimens are not reported here).

### Laboratory Tests

All specimens were processed in the laboratory of the Department of Microbiology, Gandaki Medical College Teaching Hospital. Bacterial isolates obtained in this study were identified on the basis of their cultural characteristics, cell morphology, gram stain reaction, and biochemical properties as described by Cheesbrough [19]. Antibiotic susceptibility tests were performed using the Kirby Bauer disc diffusion method and interpreted according to the Clinical and Laboratory Standard Institute (CLSI) guidelines (Annapolis Junction, MD, USA) [20]. Multidrug resistance (MDR) was defined as nonsusceptibility to at least 1 antimicrobial agent in at least 3 antimicrobial categories specific to the organisms [21].

### Determination of Cause of Death

For each case, the cause of death was determined by a panel of subject experts (internal medicine consultant, clinical pathologist, clinical microbiologist, public health specialist, and forensic pathologists). The *International Classification of Diseases, Tenth Revision, Clinical Modification*, along with the causal chain was followed while assigning cause of death to each case [22, 23].

### Statistical Analyses

Data were entered into Microsoft Excel, coded, and uploaded into SPSS version 16.0 to calculate frequencies and mean values. STATA 15.1 was used for risk factor analysis. Univariate logistic regression was used to estimate crude odds ratios (ORs), while multivariate logistic regression was used to estimate adjusted ORs to analyze the association between exposure variables and outcome (MDR *Klebsiella* infection). While estimating adjusted ORs, we dropped some exposure variables such as “chronic alcohol use” and “chronic smoking” due to collinearity. A *P* value  < .05 was considered statistically significant.

### Ethics

Informed consent was taken from the case’s family member or relative. Ethical approval was obtained from the Nepal Health Research Council Ethical Review Board. Institutional approvals were received from the management units of each study site.

## RESULTS

**Figure 1.**
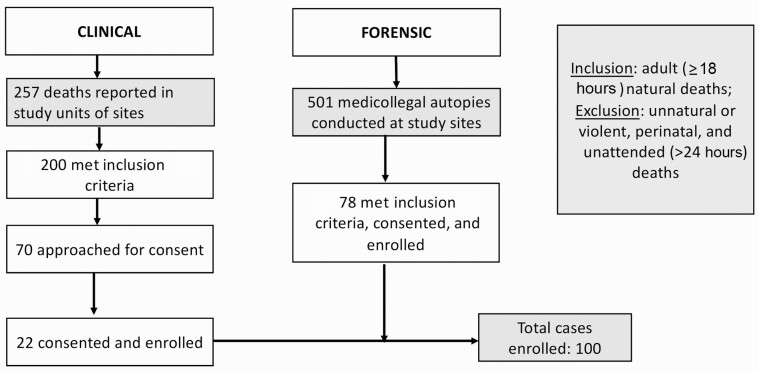
Study flow diagram (Determining Efficiently the Cause of Death among Adults and Generating Mortality Evidence at MITS Alliance Unit Nepal).

Of 257 clinical deaths screened, 200 met the inclusion criteria and 70 were approached for enrollment, of which 22 (31.4%) consented; the consent rate for eligible forensic cases (78) was 100% (Figure 1). Of the total 100 cases studied, the majority (76%) were male. The mean age of the study cases was 50.8 years (standard deviation, 15.9), with most deaths occurring among those aged 18–45 years (44%). Of all cases studied, 76 were community deaths and 24 were hospital deaths. At least 1 comorbidity was reported in 26% of cases, which was higher in the community (21%) than in the hospital (10%) deaths. Overall, 25 cases had a history of antibiotic exposure within the prior 30 days, which included all hospitalized cases and just 1 community death. The characteristics of study cases are shown in detail in Table 1.

**Table 1.** Characteristics of Reported Community and Hospital Deaths

Characteristic	Overall (N = 100)	Community Deaths (n = 76)	Hospital Deaths (n = 24)
	Frequency (Equals %)	Frequency (%)	Frequency (%)
Age, years			
18–45	44	34 (44.7)	10 (41.7)
46–65	39	30 (39.5)	9 (37.5)
>65	17	12 (15.8)	5 (20.8)
Mean age (standard deviation)	50.8 (15.9)	50.4 (15.8)	52.1 (16.5)
Gender			
Male	76	57 (75.0)	19 (79.2)
Female	24	19 (25.0)	5 (20.8)
Alcohol use (chronic)	73	57 (75.0)	16 (66.7)
Smoking (chronic)	62	54 (71.1)	8 (33.3)
Charlson comorbidity score			
0	60	47 (61.8)	13 (54.2)
1	24	14 (18.4)	10 (41.7)
≥2	2	2 (2.6)	-
Unknown	14	13 (17.1)	1 (4.2)
Hypertension	16	9 (11.8)	7 (29.2)
Tuberculosis^a^	5	4 (5.3)	1 (4.2)
Days of treatment before death			
0	76	74 (97.4)	2 (8.3)
1–7	17	1 (1.3)	16 (66.7)
8 or more	7	1 (1.3)	6 (0.25)
Antibiotic exposure in past 30 days			
No	75	75 (98.7)	-
Yes	25	1 (1.3)	24 (100.0)

^a^Of 5 cases with underlying pulmonary tuberculosis (PTB) infection, 3 from the community death group and 1 from the hospital death group were diagnosed post-mortem using the minimal invasive tissue sampling procedure. GeneXpert was performed on lung tissue specimens to detect *Mycobacterium tuberculosis* and rifampicin resistance. None of the PTB cases were rifampicin-resistant.

**Figure 2.**
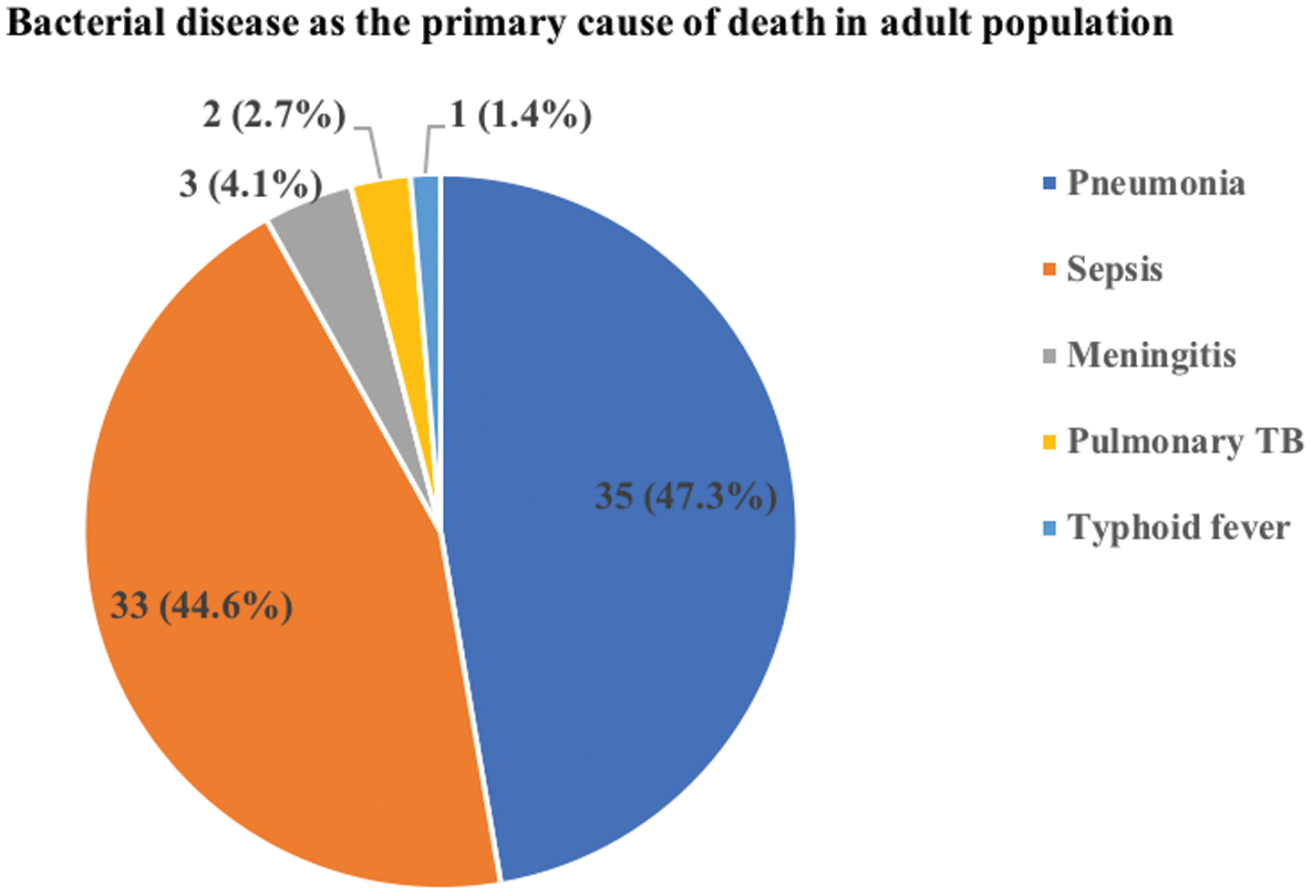
Serious bacterial diseases attributed as primary cause of death in adults (n = 74). Abbreviation: TB, tuberculosis.

At least 1 bacterial agent was detected by MITS in 86 cases. Bacterial pneumonia (72, 83.7%) was the most common infectious disease directly causing (35 of 72) or contributing (37 of 72) to death. Of these, the majority (80.5%) were from the community and only one-fourth (24.7%) were from the hospital. Among 74 cases with bacterial disease as the primary cause of death (part Ia in the causal chain), the most common were pneumonia (47.3%), sepsis (44.6%), and meningitis (4.1%), followed by pulmonary tuberculosis (2.7%) and typhoid fever (1.4%), as shown in Figure 2.

Deaths due to pneumonia and sepsis occurred more commonly in the community than in the hospital (57.1% vs 42.9% and 87.9% vs 12.1%, respectively), as shown in Table 2. *Klebsiella* species were the most common organisms causing these diseases, followed by *Proteus* species (pneumonia) and *Escherichia coli* (sepsis). Among 3 deaths due to bacterial meningitis, 2 occurred in the hospital setting caused by *Klebsiella oxytoca* and *Proteus vulgaris*.

**Table 2.** Pneumonia, Sepsis, and Meningitis as Primary Causes of Death in the Adult Population

Bacterial Disease and Causative Organism	Total	Community Deaths (n = 74)	Hospital Deaths (n = 26)
Pneumonia	35	22 (57.1%)	13 (42.9%)
*Klebsiella* species	17	10	8^a^
*Acinetobacter* species	4	1	3
*Citrobacter* species	2	2	-
*Pseudomonas aeruginosa*	3	2	1
*Proteus* species	4	4	-
*Enterobacter faecalis*	1	1	-
*Staphylococcus aureus*	2	2	1
Sepsis	33	29 (87.9%)	4 (12.1%)
*Klebsiella* species	20	18^a^	2
*Acinetobacter* species	3	2	1
*Citrobacter* species	2	2	-
*Escherichia coli*	3	3	-
*Proteus* species	3	2	1
*P. aeruginosa*	1	1	-
*S. aureus*	1	1	-
Meningitis	3	1 (33.3%)	2 (66.7%)
*Klebsiella oxytoca*	1	-	1
*E. coli*	1	-	1
*Proteus vulgaris*	1	1	-

Abbreviation: -, denotes a null value.

^a^One pneumonia and 3 sepsis cases had polymicrobial infection.

A total of 154 bacterial isolates were detected from 86 death cases; 118 (76.6%) from the community and 36 (23.4%) from the hospital. The majority of isolates were detected from lung tissue (95, 61.7%) and blood (52, 33.8%). Seven isolates were grown in CSF culture. Overall, the most common isolate was *Klebsiella* species (42.2%) followed by *Proteus* species (13.6%) among gram-negative bacilli and *S. aureus* (16.2%) among gram-positive bacilli. The variety of bacterial isolates detected in specimens (blood, CSF, and lung tissue) collected from the study cases, disaggregated by community and hospital deaths, are shown in Table 3.

**Table 3.** Bacterial Isolates Grown by Culture of Blood, Cerebrospinal Fluid, and Lung Tissue Specimens Collected from Community and Hospital Deaths

	Total		Community Deaths				Hospital Deaths			
Organism^a^	N = 154 (%)	Blood (n = 42)	CSF (n = 3)	Lung Tissue (n = 73)	Total (118)	Blood (n = 10)	CSF (n = 4)	Lung Tissue (n = 22)	Total (n = 36)
Gram-negative rods	122 (79.2)								
*Klebsiella* species	65 (42.2)	18	1	33	52	2	-	11	13
*Pseudomonas aeruginosa*	4 (2.6)	1	-	2	3	-	-	1	1
*Acinetobacter* species	11 (7.1)	3	-	2	5	2	-	4	6
*Citrobacter* species	10 (6.5)	2	-	8	10	-	-	-	0
*Escherichia coli*	7 (4.5)	3	1	-	4	1	1	1	3
*Enterobacter* species	3 (1.9)	-	-	3	3	-	-	-	0
*Salmonella* species	3 (1.9)	1	-	-	1	2	-	-	2
*Proteus* species	21 (13.6)	3	1	13	17	1	-	3	4
Gram-positive cocci									
*Staphylococcus aureus*	25 (16.2)	9	-	11	20	2	1	2	5
*Staphylococcus epidermidis*	3 (1.9)	2	-	-	2	-	1	-	1
Coagulase negative *Staphylococcus*	1 (0.6)	-	-	1	1	-	-	-	0
Gram-negative diplococci									
*Neisseria* species	1 (0.6)	-	-	-	0	-	1	-	1
Multidrug-resistant	26 (16.8)	4	-	7	11	3	1	11	15

Abbreviations: -, denotes a null value; CSF, cerebrospinal fluid.

^a^ *Klebsiella* species included *K. pneumonia* and *K. oxytoca; Citrobacter* species included *C. frundei* and *C. koseri; Enterobacter* species included *E. fecalis* and *E. aerogens; Salmonella* species included *S. typhi* and *S. paratyphi; Proteus* species included *P. vulgaris* and *P. miribilis.*

Of 154 bacterial isolates grown, 26 (16.8%) were found to be MDR; 11 detected in specimens from community deaths and 15 from hospital deaths. MDROs were also predominantly isolated from lung tissue (18 of 26) and blood (7 of 26). The only MDR isolate detected in CSF was *Neisseria* species in a chronic alcoholic patient who was admitted to the intensive care unit for 12 days and died there. An MDR isolate of *K. oxytoca* was also detected in the lung tissue of the same case.

**Figure 3.**
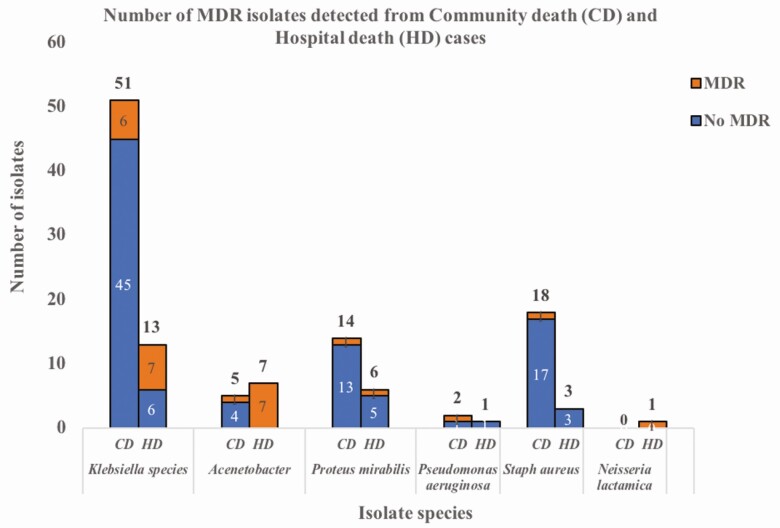
Total and MDR bacterial isolates detected in adult death cases disaggregated by CD and HD. Of the 13 MDR *Klebsiella* species included, 7 were *K. pneumoniae* and 6 were *K. oxytoca*. Abbreviations: CD, community death; HD, hospital death; MDR, multidrug-resistant.

Of all MDROs, *Klebsiella* species were the most common (50%, 13 of 26) followed by *Acinetobacter* species (30.8%, 8 of 26; Figure 3). There were 24 isolates of MDR gram-negative rods (GNRs), which included 7 isolates of *Proteus mirabilis* and 1 isolate of *P. aeruginosa*.

In our univariate analysis, MDR infection by *Klebsiella* species was found to be more likely among hospital deaths than community deaths (OR, 6.0; 95% confidence interval [CI], 1.37–26.24; *P* = .017), as shown in Table 4. We have moderate evidence against the null hypothesis that there was no difference in the odds of MDR *Klebsiella* infection occurring in males or females (*P* = .048). However, there was no association of age category with the outcome. After adjusting for confounding variables, we found that the odds of infection with MDR *Klebsiella* species was almost 23-fold higher (CI, 2.4–213.5; *P* = .006) among cases who used an antibiotic in the prior 30 days compared with those who did not use an antibiotic in prior 30 days.

**Table 4.** Association Between Risk Factors and Multidrug-Resistant Isolates of *Klebsiella* Species

Risk Factor	Non-MDR (n = 36)	MDR (n = 11)^a^	Crude OR (95% CI)	P Value	Adjusted OR (95% CI)	P Value
Age, years						
18–45	20	5	ref		ref	
46–65	11	4	1.45 (0.32–6.60)	.626	1.89 (0.30–11.82)	.493
>65	5	2	1.60 (0.24–10.80)	.630	1.26 (0.10–15.01)	.856
Gender						
Female	8	6	ref		ref	
Male	28	5	0.23 (0.06–0.99)	.048	0.07 (0.007–0.67)	.0021
Prior antibiotic use within 30 days						
No	30	4	ref		ref	
Yes	6	7	8.75 (1.93–39.57)	.005	22.85 (2.45–213.54)	.006
Chronic smoking						
No	5	7	ref			
Yes	31	4	0.09 (0.02–0.43)	.003		
Death						
Community	30	5	ref			
Hospital	6	6	6.0 (1.37–26.4)	.017		

Abbreviations: CI, confidence interval; MDR, multidrug-resistant; OR, odds ratio.

Total number of cases with *Klebsiella* species, either *K. pneumoniae* or *K. oxytoca*, detected either in blood or lung tissue or both.

^a^Two cases had MDR *Klebsiella* species in both blood and lung tissue.

## Discussion

The MITS procedure has been commonly used to determine cause of neonatal and child deaths [24-26]. Our mortality surveillance study used the MITS procedure to unravel the cause of death in an adult population and found a high incidence (86%) of bacterial infections, with serious bacterial diseases (pneumonia, sepsis, and meningitis) attributable as the primary cause of death in most (71 of 77) cases. Although most infections were detected in the community, the incidence of MDROs was found to be higher in the hospital setting (15 of 26) than in the community setting (11 of 26). Our study findings support the recommendation of global scientists that countries should monitor and report all hospital admissions and deaths attributable to drug-resistant infections [27].

GNRs are among the major culprits for causing severe hospital-acquired infections, leading to high mortality rates among both children and adults, as these organisms are highly resistant to commonly used antibiotics [28, 29]. In our study, as high as 89% (31 of 35) of deaths due to invasive pneumonia, 97% (32 of 33) of deaths due to sepsis, and all 3 deaths due to bacterial meningitis were caused by GNRs. Other studies have found that older adults are more likely to have gram-negative invasive pneumococcal disease and meningitis than other age groups [6, 30].

The incidence of MDR gram-negative bacterial infection was found to be 24 per 100 adult death cases. Similar findings have been reported by single-center studies conducted in Nepal [31, 32]. Another study conducted among 137 adult patients admitted to the intensive care unit of a tertiary center in Nepal reported 47 MDR infections per 100 patients, with an in-hospital mortality rate of 38% [33]. Among GNRs, ESBL-producing *Klebsiella* species and *Acinetobacter* species were found to be the most common MDROs in our study and in other studies from Nepal [31, 33]. The former are often resistant against β-lactam antibiotics, carbapenems, or even polymyxins and thus are responsible for high mortality, extended hospitalization, and high healthcare expenses [8, 34]. In our study, individuals with a history of prior antibiotic exposure were more likely to have MDR *Klebsiella* infection than those without such exposure history. Researchers have hypothesized that ethnicity and geographical distribution may also play a role for higher incidence of drug-resistant *Klebsiella* infection, especially among people of Asian descent [35]. The high incidence of *Acinetobacter* species, which are resistant to most of the available antimicrobial agents, is another huge concern in our setting and elsewhere [28, 36].

The high rate of mortality, coupled with less antibiotic use in the community, as opposed to excessive use of antibiotics in the hospital, could mean that people have limited access to primary care management of common bacterial infections such as pneumonia [9]. Considering this unique local circumstance, there is a need to balance improved access to antibiotics and overuse of antibiotics in order to avoid worse outcomes of serious bacterial infections and AMR [37, 38]. Moreover, an improved recording of infection episodes at the community level could inform better antibiotic stewardship [39].

As the world faces a silent pandemic of drug-resistant infections, scientists have realized the importance of the One Health concept, which signifies the linkage between human health and animal health, both of which share a common environment and community [9, 40]. Drug-resistant organisms originate from the environment and spill over to humans; therefore, AMR control initiatives should include well-coordinated and sustained surveillance systems. Understanding the evidence around relationships between antibiotic use and drug resistance would further guide the understanding of MDRO’s evolution and development of effective treatment guidelines [38].

The main strength of our study is the introduction of mortality surveillance research in Nepal, the first of its kind, to generate evidence on burden of disease at the provincial level. The study successfully implemented MITS methods to collect human specimens for laboratory processing, which is a novel concept but globally accepted method of ascertaining cause of death. Clinical as well as socioepidemiological data of mortality cases could also be collected as a part of this study, which helps stakeholders design disease prevention strategies. However, our study has a few limitations. First, it suffers from small sample size as the target number of cases assigned by the MITS Surveillance Alliance project for Nepal was only 100. Second, the study was an observational, cross-sectional, pilot study; therefore, a comparison group was absent; MDR-related findings would have been more valid if death cases were compared with individuals who survived. Moreover, there is comparison to similar studies in which deaths were not suspected to be due to infection (eg, traumatic deaths) to address the issue of contamination. Third, we could not perform molecular and genomic analyses of specimens to specify bacterial strains because this was beyond the scope of the study. Finally, case enrollment had to be halted from March 2020 to July 2020 due to COVID-19 lockdown and the government’s dead body management regulations.

## Conclusions

Mortality surveillance using the MITS technique could be a standard tool to track the burden of infectious diseases in LMICs. High incidence of serious bacterial infections as the primary cause of death among an adult population in Nepal, with most infections occurring in the community, calls for robust community surveillance mechanisms and infection prevention activities. Likewise, the high incidence of MDROs isolated from the hospitalized cases requires an effective infection management and evidence-driven antibiotic stewardship campaign in hospitals. There is a need for a multisectoral approach when adapting the One Health concept in order to control the alarming rise of AMR. Moreover, evidence generation through larger longitudinal clinical research and robust surveillance mechanisms are required to generate data and evidence that can inform infection prevention policies and clinical–public health interventions.

## Notes

***Acknowledgments.*** We acknowledge the MITS Surveillance Alliance Team for their support and guidance provided to the research team throughout the study period. We thank all contributors of the project, particularly the staff and the hospital management of all study sites, external reviewers, and experts, including Mukesh Mallik (statistical analysis). We also appreciate the cooperation of the family members and relatives of the study cases.

***Supplement sponsorship.*** This supplement is sponsored by MITS Surveillance Alliance Secretariat, led by RTI International, with funding from the Bill and Melinda Gates Foundation.

***Financial support.*** This work was supported by the MITS Surveillance Alliance. This work was supported, in whole or in part, by the Bill & Melinda Gates Foundation (OPP1180554). Payment was made to Gandaki Medical College Teaching Hospital and Research Center, Pokhara, Gandaki Province, Nepal. Under the grant conditions of the foundation, a Creative Commons Attribution 4.0 Generic License has been assigned to the Author Accepted Manuscript version that might arise from this submission.

***Potential conflicts of interest.*** All authors: No reported conflicts of interest. All authors have submitted the ICMJE Form for Disclosure of Potential Conflicts of Interest. Conflicts that the editors consider relevant to the content of the manuscript have been disclosed.

***Data availability.*** Additional data and information about study are available upon reasonable request to the researchers.
